# One year refractive outcomes of Femtosecond-LASIK in mild, moderate and high myopia

**DOI:** 10.22336/rjo.2017.5

**Published:** 2017

**Authors:** Bogdana Tabacaru, Horia Tudor Stanca

**Affiliations:** * ”Prof. Dr. Agrippa Ionescu” Emergency Hospital, Bucharest, Romania; ** “Carol Davila” University of Medicine and Pharmacy, Bucharest, Romania; *** ”Metropolitan” Hospital, Bucharest, Romania

**Keywords:** Femtosecond-LASIK, FemtoLASIK, Refractive Surgery, Myopia

## Abstract

**Purpose:** To evaluate the safety, efficacy, predictability and stability for a cohort of myopic eyes treated by Femtosecond-LASIK procedure.

**Methods:** 60 eyes (36 patients) with different degrees of myopia that underwent refractive surgery by using the Femtosecond-LASIK technique were prospectively evaluated for 12 months. The mean preoperative spherical equivalent value was –3.827 ± 1.410 diopters (D) (range: –8.125 to –1.375 D). VisuMax® femtosecond laser was used for cutting the corneal flap and then the Mel80® excimer laser for the stromal ablation.

**Results:** Mean age was 30.80 ± 5.745 years (range: 21 to 46 years) with 75% female patients. Postoperative spherical equivalent at 12 months was within ±0.25 D of emmetropia in 90% of the eyes and within ±0.50 D of emmetropia in 100% of the eyes. All the eyes achieved an uncorrected distance visual acuity (UDVA) of 1.0 (decimal scale). No eye lost lines of preoperative corrected distance visual acuity (CDVA). No major intraoperative or postoperative complications were encountered.

**Conclusions**: Femtosecond-LASIK seems to be a suitable option for the correction of mild, moderate, and high myopia, as the procedure showed to be safe, effective, and predictable for the treatment of myopic refractive errors.

## Introduction

Femtosecond Laser-Assisted In Situ Keratomileusis (FemtoLASIK) is a modern method for the correction of refractive errors, being introduced in our country for the first time in September 2011. The procedure requires two lasers, a femtosecond laser (wavelength in infrared light at 1043 nm [**1**]) for flap creation and an excimer laser (wavelength in ultraviolet light at 193 nm [**2**]) for refractive ablation. 

The purpose of our study was to evaluate the safety, efficacy, predictability, and stability for a cohort of myopic eyes treated by FemtoLASIK procedure.

## Patients and Methods

66 eyes (40 patients) were treated for mild, moderate, and high myopia by FemtoLASIK technique. All the surgeries were performed between September 1, 2011 and October 31, 2015. After being informed about the benefits and risks of the procedure, all the patients signed an informed consent.

Inclusion criteria for surgery were the patients’ wish of not wearing glasses for nearsightedness, age of 20 years or over, good general health, stable refraction for at least two years before surgery, no previous ocular trauma or ocular surgery, no ocular diseases. Exclusion criteria for surgery were estimated residual thickness of the stromal bed of less than 300 μm after the treatment, evidence, or suspect of keratoconus, severe dry eye syndrome, pregnancy, lactation, general diseases, and poor compliance. When the fundus examination revealed at-risk peripheral lesions, FemtoLASIK surgery was delayed until photocoagulation treatment was performed. 

Patients had to discontinue contact lens wearing for two weeks prior to all the corneal investigations and then again, for two weeks before the surgery.

The preoperative ocular examination included uncorrected (UDVA) and corrected distance visual acuity (CDVA), manifest and cycloplegic refraction, keratometry, anterior segment slit-lamp biomicroscopy and mydriatic fundoscopy, noncontact tonometry, corneal pachymetry, corneal topography and white-to-white (WTW) corneal diameter. 

The surgical attempted result was emmetropia in all cases. The Femtosecond laser (VisuMax®, Carl Zeiss Meditec, Germany) treatment was done first. The corneal flap thickness varied between 100 to 120 μm and the hinge was located superiorly. After the flap dissection, an excimer laser (MEL® 80, Carl Zeiss Meditec, Germany) treatment was performed. The mean ablation depth was 57.27 ± 23.032 μm (range: 21 to 130 μm). After laser ablation, a plano soft contact lens was applied.

The flap position was checked at the slit-lamp 30 minutes after the surgery in all patients.

The first appointment was in the first day postoperative when the bandage contact lens was removed. Next follow-up visits were at one month, three months, six months, and one year. UDVA and CDVA, manifest refraction and noncontact tonometry were measured and slit-lamp examination was performed at each visit. Topographies were performed at one month, six months, and one year.

Twelve patients who underwent FemtoLASIK for mild, moderate, and high myopia in one eye were also treated in the fellow eye but for compound myopic astigmatism.

Patient data were stored into an Excel® database (version 14.0, Microsoft Corp.). Statistical analysis was performed by using SPSS statistical software (version 20, IBM® SPSS® Statistics, IBM Corp.).

After testing the normality of continuous variables distributions with the Shapiro-Wilk test, statistical analysis evaluated the postoperative outcomes based on the Paired-Samples T-Test or the Wilcoxon Signed-Rank Test.

The statistically significance level was fixed at P-value ≤ 0.05.

## Results

As three patients who were treated in both eyes were lost to follow-up after the first month postoperative visit, our study included 60 eyes (31 right eyes and 29 left eyes) from 36 patients (27 females and 9 males). The mean age of included patients was 30.80 ± 5.745 years (range: 21 to 46 years). 

Visual acuity

Preoperative CDVA was 1.0 (decimal scale) in all eyes.

At the one year postoperative visit, the UDVA of 1.0 (decimal scale), equivalent to the preoperative CDVA, was obtained in all eyes. The efficacy index [**3**,**4**] (postoperative UDVA/ preoperative CDVA) was 1. The safety index [**3**,**4**] (postoperative CDVA/ preoperative CDVA) was also 1. No eye lost lines of preoperative CDVA.

Refraction

The preoperative manifest and cycloplegic refraction data are presented in **Table 1**. 

**Table 1:** Preoperative manifest and cycloplegic refraction data of the 60 myopic eyes before FemtoLASIK surgery

Manifest sphere (D)	–3.629 ± 1.397 (range: –7.75 to –1.00)
Manifest cylinder (D)	–0.395 ± 0.212 (range: –1.00 to 0)
Manifest spherical equivalent (D)	–3.827 ± 1.410 (range: –8.125 to –1.375)
Cycloplegic sphere (D)	–3.254 ± 1.448 (range: –7.50 to –1.00)
Cycloplegic cylinder (D)	–0.362 ± 0.192 (range: –0.75 to 0)
Cycloplegic spherical equivalent (D)	–3.435 ± 1.452 (range: –7.875 to –1.125)
	*D=diopters;

As the cylinder value was low and the spectacle corrections for CDVA and laser treatment were performed only with spherical diopters, our statistical analysis was focused on the manifest spherical equivalent.

Comparing the preoperative manifest and the cycloplegic mean spherical equivalent refraction data, we concluded that there was a statistically significant difference from –3.827 ± 1.410 D to –3.435 ± 1.452 D (P<0.0005) according to the paired samples t-test, the hyperopic shift being 0.391 ± 0.468 D. In order to determine the relationship between the preoperative manifest and the cycloplegic mean spherical equivalent refraction, we performed a Pearson product-moment correlation and we found a positive correlation which was statistically significant (r=0.947, P<0.0005). The refractive data for laser ablation were set according to the manifest refraction, cycloplegic refraction, and spectacle correction for CDVA.

The refractive outcomes data reported below are in accordance with the Standard Reporting in Refractive Surgery [**3**,**4**]. 

**Fig. 1:**
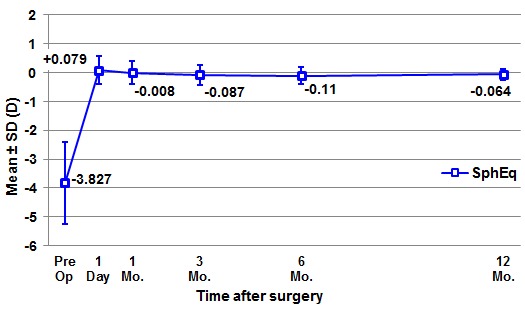
Stability – Mean change in manifest spherical equivalent during one year after FemtoLASIK surgery for the 60 myopic eyes of our study

**Fig. 1** shows the evolution of manifest refraction (spherical equivalent) after the surgery and its stability in time. 

The mean preoperative manifest spherical equivalent significantly decreased in the first postoperative day. Comparing refractive parameters as pairs of successive postoperative visits, we found no statistically significant difference until the 1-year follow-up, meaning that the spherical equivalent refraction remained stable (**Table 2**).

**Table 2:** Mean values ± SD of the manifest spherical equivalent (shown in diopters) preoperatively, and at one and 12 months after FemtoLASIK surgery, for the 60 myopic eyes of our study. P-values represent the statistical significance of the difference between two consecutive visits.

Manifest Spherical Equivalent	Diopters (mean±SD)	P-value a
Preoperative	–3.827 ± 1.410	
Postoperative 1 day	+0.079 ± 0.486	<0.0005b
Postoperative 1 month	-0.008 ± 0.394	0.247b
Postoperative 12 months	–0.064 ± 0.186	0.236c
a Statistical significance of the difference when compared to the previous visit		
b Wilcoxon Signed Rank Test		
c Paired Samples T-Test		

**Fig. 2:**
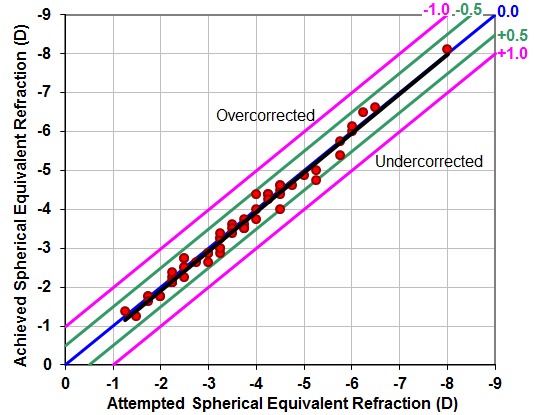
Predictability – Attempted versus achieved graph at 12 months after the FemtoLASIK surgery, for the 60 myopic eyes of our study

In order to analyze the predictability of the refractive results, we considered the attempted spherical equivalent refraction and the achieved spherical equivalent refraction. The Sperman’s correlation coefficient showed a strong correlation (r=0.971, P<0.0005). **Fig. 2** shows the linear correlation, based on the following formula: achieved spherical equivalent refraction = 0.1214 + 1.0158 * attempted spherical equivalent refraction (r2=0.943).

**Fig. 3:**
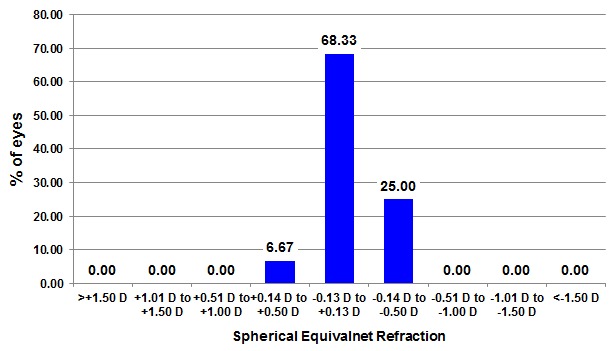
Accuracy – Spherical Equivalent Refraction to the Intended Target (D) at 12 months after the FemtoLASIK surgery, for the 60 myopic eyes of our study

The accuracy of postoperative 1-year spherical equivalent refractions compared to the intended preoperative spherical equivalent refractions is shown in **Fig. 3**. Postoperative spherical equivalent values 12 months after surgery were within ±0.25 D and ±0.5 D of emmetropia in 90% and 100% of the eyes, respectively.

Corneal Thickness

The preoperative corneal thickness was 547 ± 25.295 µm (range: 499–619 µm). It significantly decreased to 488.90 ± 28.208 µm (range: 415–554 µm) at one month postoperatively (P<0.0005, paired samples t-test) and afterwards did not significantly change at 12 months postoperatively (P=0.451, paired samples t-test).

Complications

Neither intraoperative events nor major postoperative complications such as flap dislocation, epithelial ingrowth, diffuse lamellar keratitis or flap melting, were encountered. After 12 months of follow-up, none of the eyes developed keratectasia. 

The aim of our study was to report the refractive outcomes after laser surgery; therefore, the adverse events as haze, night visual disturbances, reduced corneal sensitivity, reduced contrast sensitivity, or dry eye syndrome were not analyzed.

## Clinical Case

**Fig. 4:**
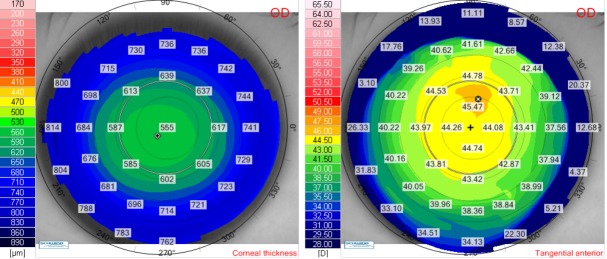
Preoperative corneal thickness map (left) and tangential anterior map (right) for the right eye of the patient V.I.C.

**Fig. 5:**
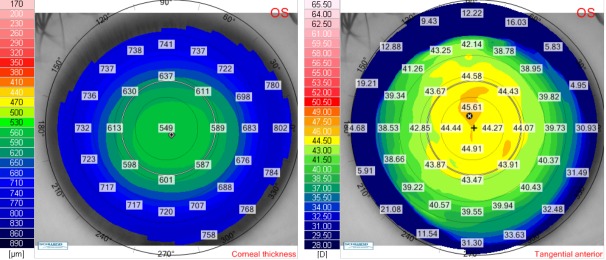
Preoperative corneal thickness map (left) and tangential anterior map (right) for the left eye of the patient V.I.C.

We presented the case of a 27-year-old female (V.I.C.), with a CDVA of 1.0 (-5.75 Dsf) in both eyes. Cycloplegic refraction for the right eye was -5.75 Dsf ^ -0.50 Dcyl x 174o and for the left eye it was -5.75 Dsf ^ -0.50 Dcyl x 10o. Keratometry for the right eye was: K flat 44.21 D x 176o and K steep 45.03 x 86o and for the left eye it was: K flat 44.50 D x 2o and K steep 44.90 x 92o. Pachymetry for the right eye was 552 μm and for the left eye, it was 547 μm. We have chosen a flap thickness of 110 μm for both eyes and an optical zone for excimer ablation of 6.5 for both eyes. The estimated residual stromal bed was 348 μm for the right eye and 343 μm for the left eye. We further presented the preoperative corneal thickness and tangential anterior maps for the right eye (**Fig. 4**) and for the left eye (**Fig. 5**).

**Table 3** (for the right eye) and **Table 4** (for the left eye) present the postoperative results, for all postoperative visits: visual acuities, manifest refraction, keratometry and pachymetry (when measured).

**Table 3:** Postoperative data for the right eye of the patient V.I.C.

Postoperative visits	Uncorrected visual acuity	Manifest refraction	K flat	K steep	Pachymetry
1 day	1.0	+0.25^-1.00 x 164o	40.00 x 170o	40.75 x 80o	-
1 month	1.0	+0.25^-0.75 x 166o	40.25 x 170o	41.00 x 80o	477 μm
3 months	1.0	+0.25^-0.50 x 171o	40.25 x 173o	40.75 x 83o	-
6 months	1.0	+0.25^-0.75 x 165o	40.25 x 167o	41.00 x 77o	478 μm
12 months	1.0	+0.25^-0.50 x 170o	40.25 x 170o	41.00 x 80o	474 μm

**Table 4:** Postoperative data for the left eye of the patient V.I.C.

Postoperative visits	Uncorrected visual acuity	Manifest refraction	K flat	K steep	Pachymetry
1 day	1.0	+0.75^-0.25 x 1o	39.50 x 4o	40.00 x 94o	-
1 month	1.0	+0.75^-0.75 x 7o	39.50 x 5o	40.75 x 95o	455 μm
3 months	1.0	+0.25^-0.50 x 0o	40.00 x 5o	40.75 x 95o	-
6 months	1.0	+0.00^-0.25 x 5o	40.25 x 8o	40.75 x 98o	460 μm
12 months	1.0	+0.25^-0.50 x 7o	40.00 x 6o	40.75 x 96o	457 μm

**Fig. 6:**
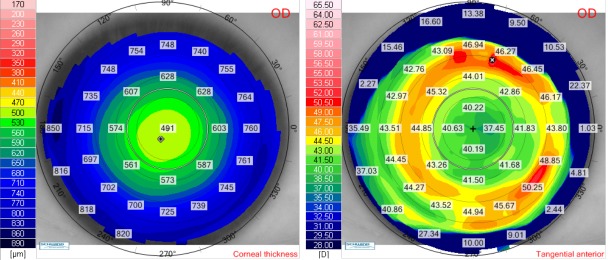
Twelve months postoperative corneal thickness map (left) and tangential anterior map (right) for the right eye of the patient V.I.C.

**Fig. 7:**
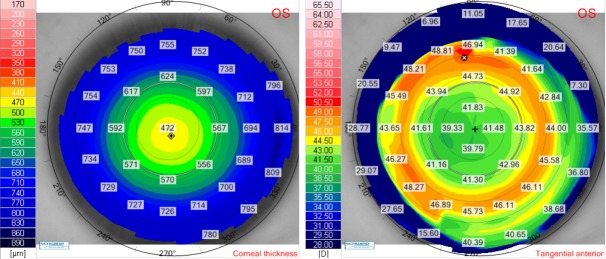
Twelve months postoperative corneal thickness map (left) and tangential anterior map (right) for the left eye of the patient V.I.C.

**Fig. 8:**
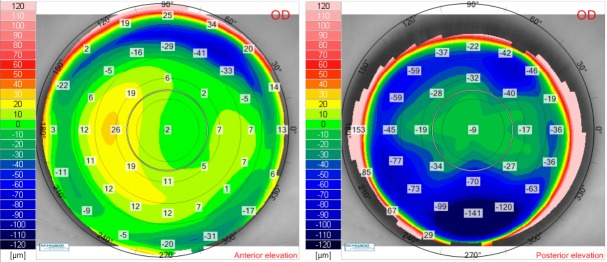
Twelve months postoperative anterior elevation map (left) and posterior elevation map (right) for the right eye of the patient V.I.C.

**Fig. 9:**
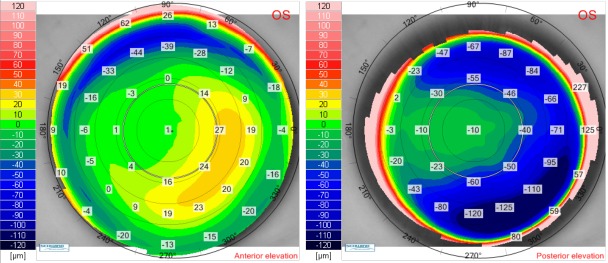
Twelve months postoperative anterior elevation map (left) and posterior elevation map (right) for the left eye of the patient V.I.C.

Postoperative corneal thickness and tangential anterior maps for the right eye (**Fig. 6**) and for the left eye (**Fig. 7**) showed a good ablation profile. Twelve months after laser surgery, there was no sign of corneal ectasia in both eyes, neither on the anterior elevation map nor on the posterior elevation map (**Fig. 8**,**9**). 

**Fig. 10:**
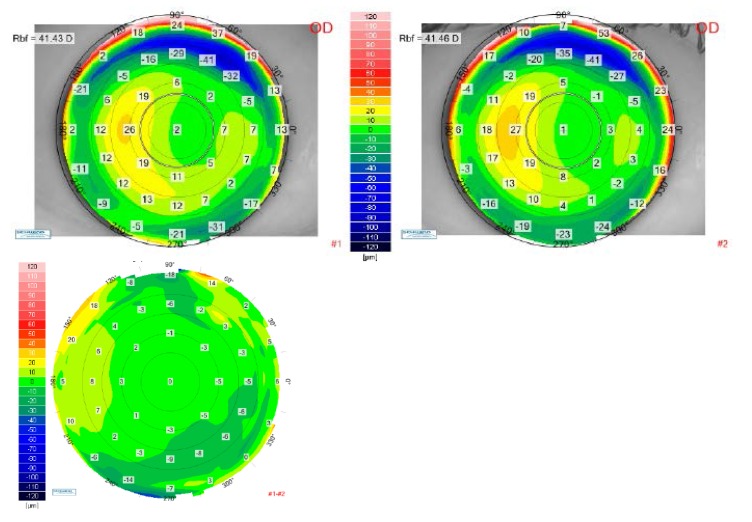
Up-left: Anterior elevation map for the right eye of the patient V.I.C. at 1-month postoperative visit. Up right: Anterior elevation map for the right eye of the patient V.I.C. at 12 months postoperative visit. Bottom: Differential anterior elevation map between postoperative 1 month and postoperative 12 months for the right eye of the patient V.I.C.

**Fig. 11:**
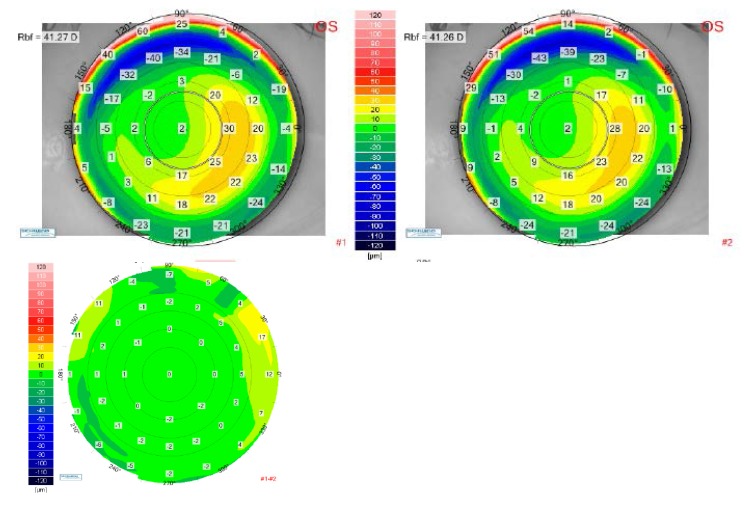
Up-left: Anterior elevation map for the left eye of the patient V.I.C. at 1-month postoperative visit. Up right: Anterior elevation map for the left eye of the patient V.I.C. at 12 months postoperative visit. Bottom: Differential anterior elevation map between postoperative 1 month and postoperative 12 months for the left eye of the patient V.I.C.

The differential anterior elevation map between the postoperative visit at 1 month and the postoperative visit at 12 months are presented in **Fig. 10**) (right eye) and **Fig. 11**) (left eye), which best show no change in the corneal shape (no risk of anterior corneal ectasia) during one year follow-up. 

## Discussion

Compared to the other nowadays techniques of correcting myopic refractive errors, Femtosecond-LASIK was comparable with Small Incision Lenticule Extraction (SMILE) [**5**-**7**] and Transepithelial Photorefractive Keratectomy (Transepithelial-PRK) [**8**] in terms of safety, efficacy and predictability. After Femto-LASIK, the corneal sensitivity was lower and the dry eye syndrome was more frequent than after SMILE [**5**] but compared to Transepithelial-PRK had a shorter recovery time [**8**]. 

Referring to the flap cutting, either the Femtosecond laser (in Femto-LASIK procedure) or the mechanical microkeratome (in LASIK procedure) demonstrated to be safe and effective in correcting myopia, with stable results and no significant difference in postoperative UCVA and CDVA [**9**]. However, the femtosecond laser may have advantages over the microkeratome in better flap thickness predictability, fewer induced high order aberrations, and longer tear break-up time [**9**]. 

To the best of our knowledge, in our country, this is the first study on Femtosecond-LASIK refractive results for myopia correction.

We achieved maximum UDVA and manifest refraction close to emmetropia at all postoperative visits with no major intraoperative or postoperative complication for all the treated eyes in our study cohort.

Our study’s weaknesses included the small number of study eyes and the short period of follow-up. According to J.Alió et al., myopic regression is possible up to 5 years of follow-up and it is correlated with the achieved correction [**10**]. Further reports will be salutary after following-up the patients for a longer period of time. 

In conclusion, Femtosecond-LASIK seems to be a suitable option to correct mild, moderate, and high myopia, as the procedure showed to be safe, effective, and predictable for the treatment of myopic refractive errors.

**Financial Disclosure**

None of the authors has any financial or proprietary interests to disclose.
